# Tspan5 promotes epithelial–mesenchymal transition and tumour metastasis of hepatocellular carcinoma by activating Notch signalling

**DOI:** 10.1002/1878-0261.12980

**Published:** 2021-07-27

**Authors:** Qian Xie, Huiling Guo, Peirong He, Huan Deng, Yanjun Gao, Ningning Dong, Wenbo Niu, Tiancai Liu, Ming Li, Suihai Wang, Yingsong Wu, Ji‐Liang Li

**Affiliations:** ^1^ Key Laboratory of Antibody Engineering of Guangdong Higher Education Institutes School of Laboratory Medicine and Biotechnology Southern Medical University Guangzhou China; ^2^ Wenzhou Medical University Eye Hospital and School of Biomedical Engineering China; ^3^ Cancer Research Centre University of Chinese Academy of Sciences Wenzhou Institute China; ^4^ Institute of Translational and Stratified Medicine University of Plymouth Faculty of Medicine and Dentistry UK

**Keywords:** ADAM10, EMT, hepatocellular carcinoma, Notch, Tspan5

## Abstract

Hepatocellular carcinoma (HCC) is one of the most lethal cancers worldwide due to a high rate of tumour metastasis and disease recurrence. In physiological conditions, tetraspanins interact with specific partner proteins in tetraspanin‐enriched microdomains and regulate their subcellular localization and function. However, the function of Tspan5 in pathological processes, particularly in cancer biology and its clinical significance, are still unclear. Here, we describe that a high expression of Tspan5 is significantly associated with some clinicopathological features including invasive length, vascular invasion, clinical stage and poor overall survival of HCC patients. Alterations of Tspan5 expression by lentivirus transductions in HCC cells demonstrated that Tspan5 promotes wound healing and cell migration *in vitro* and tumour metastasis of HCC cells *in vivo*. Mechanistic studies revealed that Tspan5 promoted cell migration and tumour metastasis by increasing the enzymatic maturation of ADAM10 and activating Notch signalling via the increase of the cleavage of the Notch1 receptor catalysed by the γ‐secretase complex. Activation of Notch signalling by Tspan5 was shown further to enhance the epithelial–mesenchymal transition (EMT) and actin skeleton rearrangement of tumour cells. In clinical HCC samples, Tspan5 expression is strongly correlated with many key molecules acting in Notch signalling and EMT, highlighting the role of Tspan5 in the regulation of Notch signalling, EMT and tumour metastasis of HCC. Our findings provide new insights into the mechanism of tumour metastasis and disease progression of HCC and may facilitate the development of novel clinical intervention strategies against HCC.

## Graphical abstract

We demonstrated that Tspan5 promotes the enzymatic maturation of ADAM10 and activates Notch signalling. The γ‐secretase complex catalyses the cleavage of Notch1 for the release of NICD, which is then translocated to the nucleus where it activates Notch target gene transcription. By activating Notch signalling, Tspan5 promoted epithelial–mesenchymal transition, actin skeleton rearrangement, tumour migration and metastasis of HCC, resulting in poor patient survival.

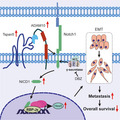

**Abbreviations**
ADAM10: active metalloprotease of the A disintegrin and metalloprotease 10
DAPI: 4,6‐diamidino‐2‐phenylindole
DBZ: dibenzazepine
EMT: epithelial–mesenchymal transition
H&E: haematoxylin and eosin
HCC: hepatocellular carcinoma
Hes5: Hes family BHLH transcription factor 5
IF: immunofluorescence
IHC: immunohistochemical staining
NICD1: Notch1 intracellular domain
qRT‐PCR: quantitative real‐time polymerase chain reaction
TCGA: The Cancer Genome Atlas
TEM: tetraspanin‐enriched microdomain

## 1 Introduction

Hepatocellular carcinoma (HCC) is one of the most common and malignant tumours, ranked as the fifth most common cancer and the third leading cause of cancer‐related death worldwide [1]. The incidence of HCC is on the rise globally, with China leading the total number of new cases and deaths [2]. There are no apparent clinical symptoms in an early stage of HCC and most cases are diagnosed at an advanced stage. Thus, the prognosis is very poor, having a 5‐year relative survival rate of approximately 18% only [3]. Currently, standard treatments include surgical resection, liver transplantation, radiofrequency ablation, chemoembolization and systemic therapy, but the high incidence of recurrence and metastasis leads to unsatisfactory outcomes [3]. Like many other types of cancer, HCC growth and metastasis can be triggered by the dominant activation of classical proto‐oncogenes and/or inactivation of tumour suppressor genes [4]. It has been reported that mutations in genes such as *CTNNB1*, *AXIN1*, *ARID1A*, *ARID2*, *NFE2L2* and *KEAP1* promote hepatocarcinogenesis and disease progression [3]. Importantly, a variety of cell signal pathways such as VEGF/VEGFR, RAF/MEK/ERK, PI3K/AKT/mTOR and Wnt/β‐catenin play crucial roles in the progression of disease, and inhibitors of such pathways, named targeted drugs, are used as potential anticancer drugs for clinical studies [5]. In fact, targeted drugs such as sorafenib, lenvatinib, regorafenib, cabozantinib, ramucirumab and nivolumab have been approved to integrate systemic therapy for HCC, but their therapeutic efficacies are far from achieving patient satisfaction [6, 7, 8, 9, 10, 11]. Therefore, better understanding of the molecular mechanisms of carcinogenesis and metastasis of HCC will facilitate the development of new intervention approaches and therapeutic targets.

Tetraspanins are evolutionarily conserved small proteins of 204–355 amino acids (20–50 kDa), characterized by four conserved transmembrane domains (TM), small and large extracellular loops (SEL and LEL), a short intracellular loop (between TM2 and TM3) and short cytoplasmic amino‐ and carboxyl‐terminal tails [12]. The interactions among tetraspanins and between tetraspanins and their specific partner proteins form tetraspanin‐enriched microdomain (TEM) [13]. Crystal structure of either CD81 (Tspan28) or CD9 (Tspan29) reveals a cone‐like architecture and a large intramembrane cavity created by four TM helices [14, 15]. Both TM3 and LEL are critical for the molecular association of tetraspanin with its partner proteins, and lipid binding to the central pocket could modulate the molecular association by affecting the LEL conformation [14, 15]. Tetraspanins are believed to regulate subcellular localizations and coordinate functions of their interactive partners including cell adhesion proteins, cell surface receptors, proteases and intercellular signalling molecules, thereby engaging in diverse molecular and cellular processes ranging from cell adhesion, migration, invasion, signalling, cell–cell fusion, infection by cancer‐causing viruses, morphology to survival during multiple stages of cancer development [16]. A total of 33 members in the tetraspanin superfamily have been identified in mammalian cells, some of which are known to regulate the migration and invasion of tumour cells, thereby manipulating the progression and metastasis of many types of cancers [16, 17]. CD9 (Tspan29), CD63 (Tspan30) and CD82 (Tspan27) inhibit metastasis of various cancers, whereas Tspan8 and CD151 (Tspan24) promote metastasis [16, 17, 18]. CD151 can interact with integrin α6β1 and α6β4 to regulate tumour growth, migration, metastasis, signal transduction and drug resistance. Tspan12 can contribute to the carcinogenicity of ADAM10 by regulating its maturation and function [19].

Tspan5 (also called NET‐4, TM4SF9) is widely distributed in various organs and tissues [12, 20]. Previous studies have shown that Tspan5 plays an important role in osteoclast formation and differentiation through the Notch pathway [21, 22]. Tspan5 interacts with ADAM10, regulates ADAM10 exit from endoplasmic reticulum and trafficking to the membrane surface, enhances the enzymatic maturation of ADAM10, and thereby increases ADAM10‐dependent Notch signalling [23, 24, 25, 26, 27]. However, the function of Tspan5 in pathological processes, particularly in tumorigenesis and metastasis, is unknown. We have been interested in the role of tetraspanins in cancer biology and clinical significance. Previously, we reported that Tspan5 is downregulated in gastric cancer tissues and functions as a tumour suppressor in stomach to control the tumour growth by regulation of cell cycle transition from G1‐S phase via decreasing the expression of cyclin D1, CDK4, pRB and E2F1 [28].

In this study, we demonstrate that the expression of Tspan5 is significantly associated with tumour invasive depth, vascular invasion, clinical stage and poor overall survival of HCC patients. Functional investigations demonstrated that Tspan5 increases the migration *in vitro* and metastasis *in vivo* of HCC cells. Mechanistic studies further revealed that Tspan5 enhances the expression of active ADAM10, activates Notch signalling, promotes the epithelial–mesenchymal transition (EMT) and triggers the tumour metastasis of HCC. Stunningly, Tspan5 is highly correlated with the expression of many key elements examined in both Notch signalling and EMT in clinical HCC samples.

## 2 Methods

### 2.1 Patients and HCC tissues

A cohort of 139 HCC tumour samples on tissue microarrays (HLiv‐HCC150CS‐01, HLiv‐HCC180Sur‐05) containing clinicopathological information were purchased from Shanghai Outdo Biotech Co., Ltd (Pudong New Area, Shanghai, China) (http://www.superchip.com.cn/). All clinical samples were categorized into gender, age, tumour size, invasive depth, metastasis, pathological grade and TNM stage. TNM stages were categorized according to the 7th edition of the American Joint Committee on Cancer Staging Manual [29]. The study methodologies conformed to the standards set by the Declaration of Helsinki. Patient consent and approval from the local Ethics Committee were obtained only for research in the use of clinical materials.

### 2.2 Cell lines and cell culture

Human normal hepatocyte (HL7702) and hepatoma cell lines (BEL7402, Hep3B, HUH7, MHCC97H, MHCC97L, PLC, QGY7701 and SK‐Hep1) were purchased from and authenticated by the Typical Culture Preservation Commission Cell Bank (Chinese Academy of Sciences, Xuhui, Shanghai, China). BEL7402 cell line was cultured in RPMI 1640 (Gibco, Grand Island, NY, USA) but Hep3B and MHCC97L cell lines were cultured in Dulbecco's modified Eagle's medium (DMEM) (Gibco), each supplemented with 10% FBS (Gibco), 100 U·mL^−1^ penicillin, and 100 µg·mL^−1^ streptomycin. All cultures were maintained at 37°C with 5% CO_2_.

### 2.3 Lentivirus transduction

The lentiviruses, packaged with the Ubi‐MCS‐3FLAG‐CBh‐gcGFP‐IRES‐puromycin vector containing a full coding region of the *TSPAN5* gene (NM_005723), were purchased from Shanghai GeneChem Co., Ltd (Zhangjiang Hi‐Tech Park, Shanghai, China). The lentiviruses, packaged with the pGPH1/GFP/Neo vector containing short hairpin RNA (shRNA) sequence targeting Tspan5 (sh1009:5′‐GCAGAAGATGTCATCAACACT‐3′) or scrambled control sequence (5′‐GTTCTCCGAACGTGTCACGT‐3′), were purchased from Shanghai GenePharma Co., Ltd (Zhangjiang Hi‐Tech Park). The viruses were transduced into HCC cell lines according to the manufacturer protocol. Puromycin (3–5 μg·mL^−1^) was used for selection of stable cell lines. The lentiviruses used for *in vivo* imaging experiments were packaged with the pLenti‐CBh‐3FLAG‐luc2‐tCMV‐tdTomato‐F2A‐blasticidin vector and purchased from Shanghai Obio Technology Co., Ltd (Pudong New Area, Shanghai, China). Blasticidin (10 μg·mL^−1^) was used for selection of stable cells. All functional experiments were conducted within 2 weeks following the lentiviral infection.

### 2.4 Western blotting

Protein extractions and western blotting were performed as described previously [30]. Primary antibodies include antibodies against Tspan5 (1 : 3000, SAB2108599, Sigma‐Aldrich, St. Louis, MO, USA), Flag‐tag (1 : 1000, F1804, Sigma‐Aldrich), E‐cadherin (1 : 1000, BF0219, Affinity Biosciences, Cincinnati, OH, USA), N‐cadherin (1 : 1000, AF4039, Affinity Biosciences), vimentin (1 : 1000, #5741, Cell Signaling Technology, Boston, MA, USA), Snail (1 : 1000, #3895, Cell Signaling Technology), ADAM10 (1 : 1000, #14194, Cell Signaling Technology), cleaved Notch1 (Val1744) (1 : 1000, #4147, Cell Signaling Technology), Hes5 (1 : 1000, ab194111, Abcam, Cambridge Biomedical Campus, Cambridge, UK) and GAPDH (1 : 5000, Bioworld Technology, Bloomington, MN, USA). GAPDH was used as a loading control to normalize the protein expression. Protein bands on the blots were visualized by ECL chemiluminescence reagent and quantified by imagej software (NIH, Bethesda, MD, USA) for densitometry analysis. Each experiment was repeated at least three times.

### 2.5 Quantitative real‐time polymerase chain reaction

RNA extraction, cDNA synthesis and quantitative real‐time polymerase chain reaction (qRT‐PCR) were performed as described [30]. Primers used for qRT‐PCR to detect Tspan5 include forward 5′‐TTGTGGTGGGAGGAGTGAT‐3′ and reverse 5′‐CTGGGTGAAGTCTATGAGGTT‐3′. Each experiment was repeated at least three times.

### 2.6 Immunohistochemistry and scoring

Immunohistochemistry (IHC) was performed as described previously [30] to analyse HCC clinical specimens and metastatic foci in lung tissues of xenografted mice with primary antibody against Tspan5 (1 : 100, SAB2108599, Sigma‐Aldrich), E‐cadherin (1 : 100, 20874‐1‐AP, ProteinTech Group, Rosemont, IL, USA), vimentin (1 : 100, #5741, Cell Signaling Technology) and NICD1 (anti‐activated Notch1) (1 : 100, ab8925, Abcam). Stained sections were analysed by using imagej software. The expression of Tspan5 protein was scored according to the mean density (the ratio of the integral optical density to the total area). High and low protein expression was defined using the mean score of all samples as a cutoff point. The relationship between Tspan5 expression and pathoclinical parameters of HCC patients was analysed by the Pearson Chi‐square test.

### 2.7 Immunofluorescence assays

Tumour cells were planted on glass coverslip in 6‐well plates at 37°C with 5% CO_2_ for 24 h and fixed with 4% paraformaldehyde for 15 min. After washing three times with PBS, cells were blocked with 5% BSA at room temperature for 90 min, followed by incubation with primary antibody in a moist chamber at 4°C overnight. To detect nuclear proteins, cultured cells were permeabilized with PBS containing 0.25% Triton X‐100 (Biyuntian, Songjiang, Shanghai, China) for 15 min before blocking. Primary antibodies include those against E‐cadherin (1 : 100, 20874‐1‐AP, ProteinTech Group), vimentin (1 : 100, #5741, Cell Signaling Technology) and NICD1 (anti‐activated Notch1) (1 : 100, ab8925, Abcam). After three washes, cells were incubated with Alexa Fluor 488 goat anti‐mouse or anti‐rabbit IgG at room temperature for 1 h. Cells were washed in PBS and incubated with 4,6‐diamidino‐2‐phenylindole (DAPI) (Biyuntian) for 10 min. The fluorescent signals from stained cells were observed using a Zeiss LSM880 confocal microscope (Carl Zeiss, Oberkochen, Germany). Fluorescent intensity representing the amount of protein expressed in tumour cells was determined by Zeiss zen microscope software.

### 2.8 Wound‐healing assays

Cells were seeded in 6‐well plates (4 × 10^5^ cells per well) in triplicate and a scratch was performed in the middle of each well with a 200‐μL pipette tip. The cells were gently washed with PBS to remove debris. After incubation for 48–96 h (dependent upon cell line), photographs were taken to estimate closure of the gap in at least three randomly selected fields. Wound closure was then calculated using the formula: wound closure (%) = (area of gap at the starting point − area of gap at the ending point)/area of gap at the starting point. Each experiment was repeated at least three times.

### 2.9 Boyden chamber cell migration assays

Effects of Tspan5 and Notch inhibitor on cell migration were measured using Boyden chambers (8‐µm pore; Corning Star, Cambridge, MA, USA). Cells in serum‐free medium (3 × 10^5^ cells per 200 µL) were added to the upper chamber of Transwell plates. Then, 0.8 mL medium supplemented with 10% FBS was added to the lower chamber. After incubation at 37°C for 6−48 h (dependent upon cell line), cells that had migrated and stuck to the lower surface of the membrane were immobilized with 100% methanol for 5 min and stained with 0.5% crystal violet for 5 min. For quantification, cells were counted under a microscope in three randomly selected fields (original magnification, 200×). Each experiment was repeated at least three times.

### 2.10 F‐actin staining and confocal microscope examinations

F‐actin staining was performed by Rhodamine‐phalloidin (5 µg·mL^−1^) and DAPI (0.1 µg·mL^−1^) as described previously [31]. The fluorescent signals from stained cells were observed using a Zeiss LSM880 confocal microscope (Carl Zeiss). Fluorescent intensity representing the amount of protein expressed in tumour cells was determined by Zeiss zen microscope software.

### 2.11 Tumour xenograft

Male BALB/c nu/nu (CAnN.Cg‐*Foxn1^nu^*/Crl) mice 4–6 weeks of age were purchased from the Experimental Animal Centre of Southern Medical University (Guangzhou, China) and maintained under standard pathogen‐free conditions. All experimental procedures were approved by the Ethical Committee of Southern Medical University. Animal welfare was closely monitored in accordance with the Guide for the Care and Use of Laboratory Animals of the National Institutes of Health. Tumour xenografts were essentially performed as described previously [32, 33]. Each group contained five to six mice. For pulmonary metastasis assay, HCC cells (4 × 10^6^ in 200 μL) were xenografted to nude mouse by tail vein injection. After 12 weeks, the mice were humanely sacrificed by neck dislocation and mouse lungs collected. Consecutive sections of the whole lung were subjected to haematoxylin and eosin (H&E) staining. The number and diameter of all metastatic foci in the lung were calculated to evaluate the development of pulmonary metastasis. For life imaging, HCC cells were infected with lentivirus containing luciferase‐encoding vector. At week 4 and week 8, D‐luciferin (15 mg·mL^−1^, 122799, PerkinElmer, Waltham, MA, USA) was injected intraperitoneally at a dose of 150 mg·kg^−1^ bodyweight. Fifteen minutes later, the mouse was imaged under general anaesthesia with the In Vivo Fx Pro Imaging System (Bruker, Billerica, MA, USA) to analyse the lung metastasis.

### 2.12 Statistical analysis

Data were shown as mean ± standard deviation (SD). The spss 20.0 software (SPSS Inc., Almonk, NY, USA) and graphpad prism 5 software (GraphPad Software Inc., San Diego, CA, USA) were used for all data analysis. The analysis of variance (ANOVA) test was used to compare mean values among three or more groups, and independent‐sample Student's *t*‐test was used to compare two groups with normal distribution data. The data normality was verified using the Kolmogorov–Smirnov test. For non‐normal distribution data, the Mann–Whitney test was used for two‐group comparisons, and the Jonckheere–Terpstra test was used to compare more than two groups. Kaplan–Meier plots and the log‐rank test were used for analysis of overall survival data. Clinical correlations were analysed by Pearson's Chi‐square test. Statistical significance was indicated by asterisks (*P < 0.05, **P < 0.01, ***P < 0.001 and ****P < 0.0001).

## 3 Results

### 3.1 Tspan5 is upregulated and correlated with clinicopathological features and overall survival of HCC patients

**Fig. 1:**
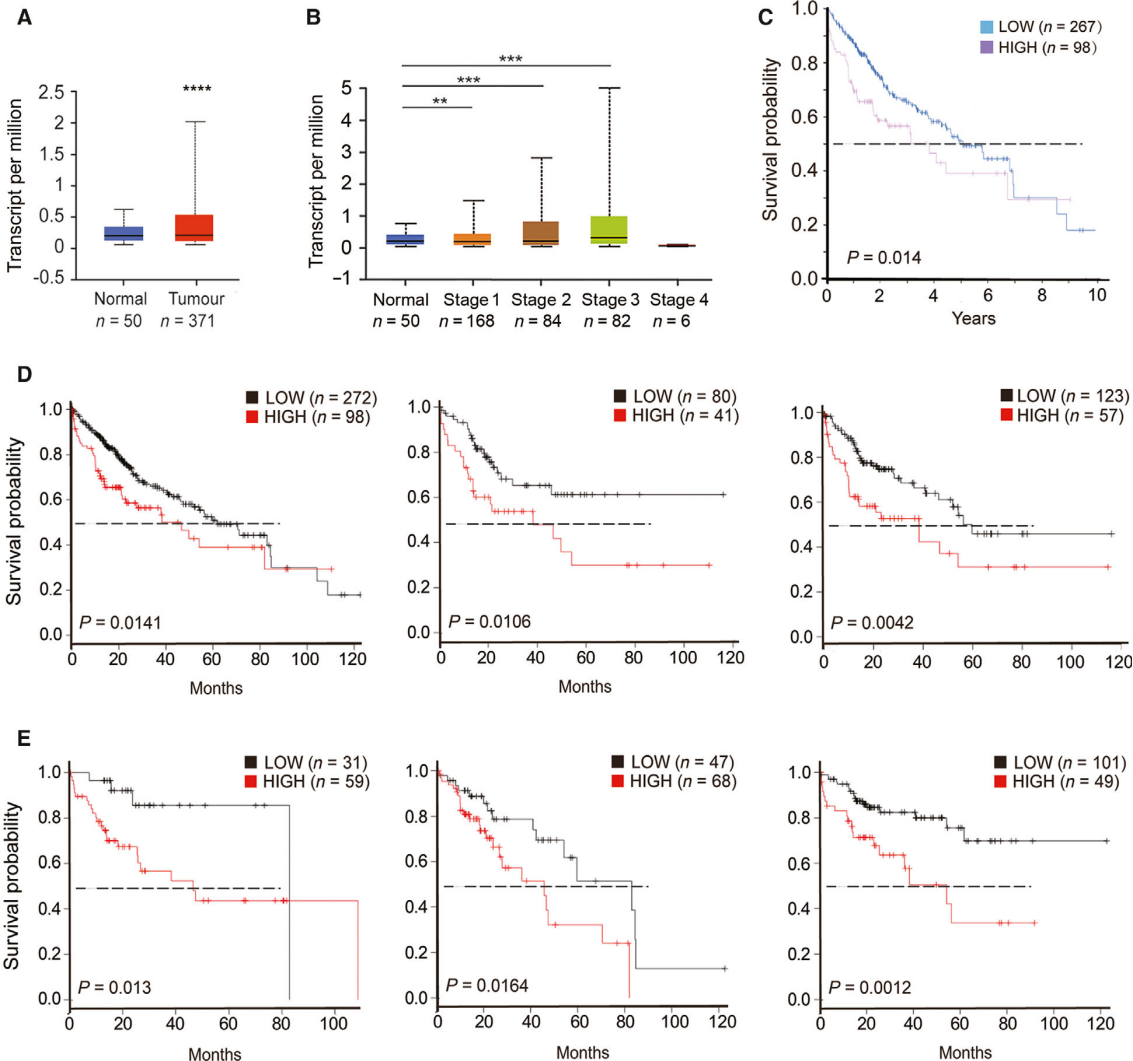
Tspan5 is correlated with clinicopathological features and overall survival of HCC patients (*n* = number of patients). (A) Expression of Tspan5 is upregulated in all HCC tissues of white, African‐American and Asian patients versus in liver normal tissues in UALCAN database (*P* = 6.46E‐8) (http://ualcan.path.uab.edu/cgi‐bin/TCGAExResultNew2.pl?genenam=TSPAN5&ctype=LIHC). Student's *t*‐test, ****P < 0.0001. The error bars indicate maximum and minimum values, respectively. (B) Expression of Tspan5 is gradually increased with individual cancer stages of HCC in UALCAN database. ANOVA test, **P < 0.01, ***P < 0.001. The error bars indicate maximum and minimum values, respectively. (C) High expression of Tspan5 is associated with poor overall survival of HCC patients (Log‐rank test, *P* = 0.014) in Human Protein Atlas (https://www.proteinatlas.org/ENSG00000168785‐TSPAN5/pathology/liver+cancer). (D) High expression of Tspan5 is associated with poor overall survival of HCC patients (Log‐rank test, *P* = 0.0141, left panel), stratified patients with pathological grade 3 (Log‐rank test, *P* = 0.0106, middle panel) and high mutation burden (Log‐rank test, *P* = 0.0042, right panel) in Kaplan–Meier plotter database (Pan‐cancer RNA‐seq) (http://kmplot.com/analysis/index.php?p=service&start=1). (E) High expression of Tspan5 is associated with poor overall survival of HCC patients with vascular invasions (Log‐rank test, *P* = 0.013, left panel), alcohol consumptions (Log‐rank test, *P* = 0.0164, middle panel) and hepatitis virus infections (Log‐rank test, *P* = 0.0012, right panel) in Kaplan–Meier plotter database (Liver cancer RNA‐seq).

We first investigated the expression of Tspan5 in HCC tissues. IHC staining for Tspan5 protein expressed in our cohort with 139 HCC clinical samples showed that Tspan5 expression was mainly located on cell membranes and in cytoplasm (Fig. S1A) and was significantly associated with invasive depth (*P* = 0.032) and TNM stage (*P* = 0.015) but not with gender, age, tumour size or pathological grade. Increased expression of Tspan5 was frequently observed in tumours that are more invasive and in a later clinical stage (Table 1). Bioinformatics analysis of Oncomine datasets showed Tspan5 transcripts in HCC were 1.2–2.6‐fold higher than those of normal liver (*P* < 0.0001) (Fig. S1B). Analysis of the UALCAN dataset revealed Tspan5 transcripts were upregulated 1.1‐fold in tumour tissues of white, African‐American and Asian patients (*P* < 0.0001) (Fig. 1A). Tspan5 transcripts were significantly associated with pathological grades (Fig. S1C) and individual cancer stages (Fig. 1B).

**Table 1:** Association of Tspan5 protein expression with clinicopathological characteristics of 139 HCC patients.

Characteristics	No. of cases	Expression of Tspan5 (%)		*P*‐values^a^
		Low	High	
Gender				
Male	123	56 (45.5)	67 (54.5)	0.419
Female	16	9 (56.2)	7 (43.8)	
Age				
< 55	74	37 (50.0)	37 (50.0)	0.414
≥ 55	65	28 (43.1)	37 (56.9)	
Tumour size (cm)				
< 5	67	34 (50.7)	33 (49.3)	0.364
≥ 5	72	31 (43.1)	41 (56.9)	
Invasive depth				
T1–T2	83	45 (54.2)	38 (45.8)	0.032*
T3–T4	56	20 (35.7)	36 (64.3)	
Pathological grade				
I–II	96	41 (42.7)	55 (57.3)	0.152
III	43	24 (55.8)	19 (44.2)	
Metastasis				
No	135	64 (47.4)	71 (52.6)	0.376
Yes	4	1 (25)	3 (75)	
TNM stage				
I–II	79	44 (55.7)	35 (44.3)	0.015*
III–IV	60	21 (35.0)	39 (65.0)	

Bold indicates statistical significance (*P < 0.05).

a Chi‐square test.

We then evaluated the association of Tspan5 expression with patient overall survival. Kaplan–Meier survival analysis showed the expression of Tspan5 transcripts in the Human Protein Atlas was inversely associated with overall survival of HCC patients (*P* = 0.014) (Fig. 1C). Further investigation of Kaplan–Meier Plotter dataset (Pan‐cancer RNA‐seq) demonstrated that high expression of Tspan5 was significantly correlated with poor overall survival of all HCC patients (*P* = 0.0141), stratified patients with pathological grade 3 (*P* = 0.0106) and high mutation burden (*P* = 0.0042) (Fig. 1D). Moreover, investigation of the Kaplan–Meier Plotter dataset (Liver cancer RNA‐seq) showed that high expression of Tspan5 was significantly correlated with vascular invasion (*P* = 0.013), alcohol consumption (*P* = 0.0164) and hepatitis virus infection (*P* = 0.0012) (Fig. 1E). These results suggest that Tspan5 may play a pivotal role in tumour metastasis of HCC and be closely associated with alcohol consumption and hepatitis virus infection.

### 3.2 Tspan5 promotes tumour cell migration *in vitro* and HCC metastasis *in vivo*

**Fig. 2:**
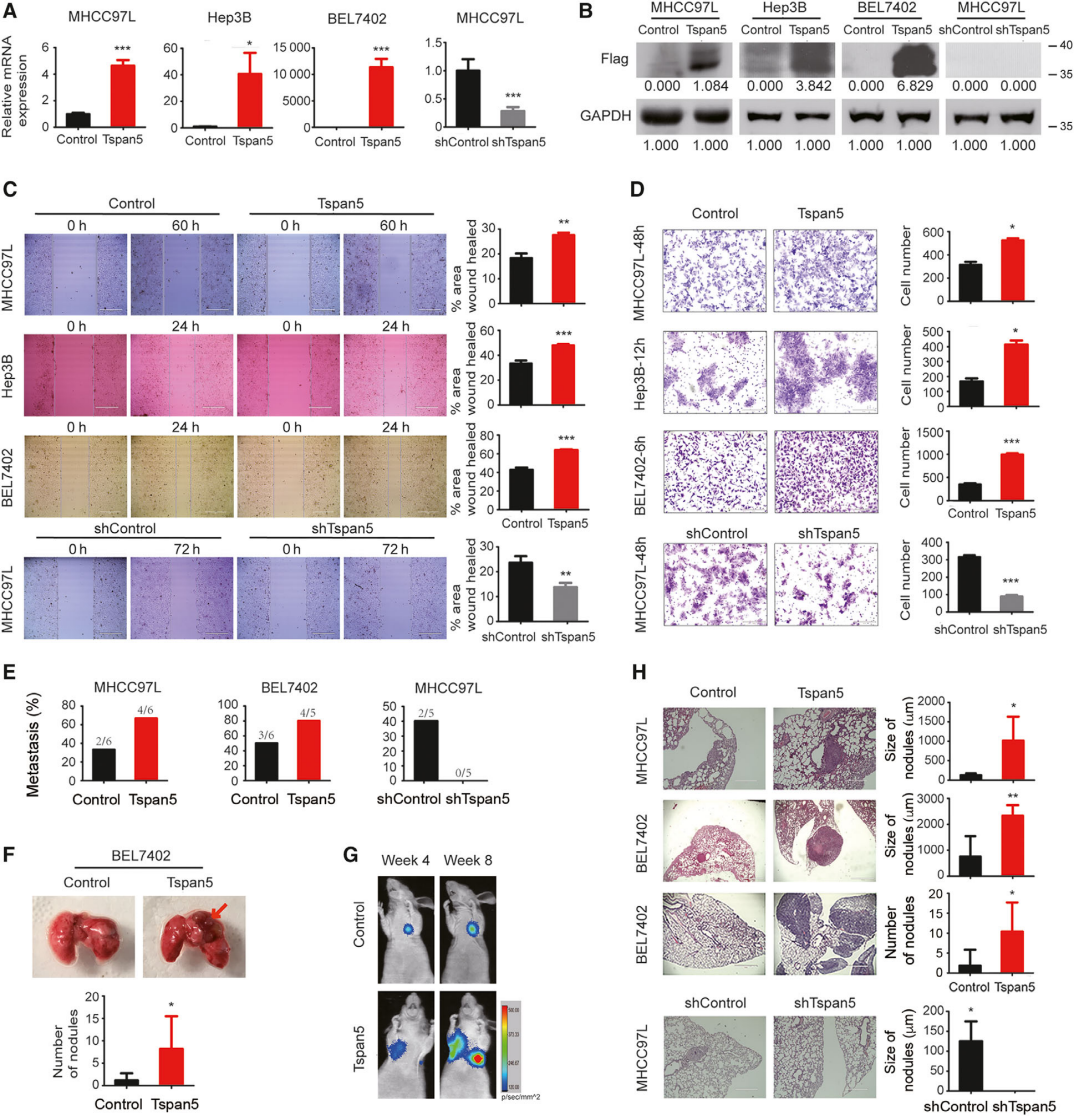
Tspan5 promotes the migration and metastasis of HCC cells. (A) Verification of Tspan5 expression at the mRNA level in MHCC97L, Hep3B and BEL7402 cell lines by qRT‐PCR. Mean ± SD. Student's *t*‐test, *n* = 3, *P < 0.05, ***P < 0.001. (B) Verification of Tspan5 expression at the protein level in MHCC97L, Hep3B and BEL7402 cell lines by western blotting. (C) Wound‐healing assays showing that upregulation of Tspan5 increases cell migration of MHCC97L, Hep3B and BEL7402 (top 3 panels), whereas downregulation of Tspan5 decreases cell migration of MHCC97L (bottom panel). Mean ± SD. Student's *t*‐test, *n* = 3, **P < 0.01, ***P < 0.001. Scale bar: 400 μm. (D) Boyden chamber cell migration assays showing that upregulation of Tspan5 increases cell migration of MHCC97L, Hep3B and BEL7402 (top 3 panels), whereas downregulation of Tspan5 decreases cell migration of MHCC97L (bottom panel). Mean ± SD. Student's *t*‐test, *n* = 3, *P < 0.05, ***P < 0.001. Scale bar: 200 μm. (E) Lung metastasis proportion of nude mice xenografted with Tspan5‐upregulated or control HCC cells via tail vein injections, *n* = 5–6 mice per group. (F) Lung morphology of nude mice xenografted with Tspan5‐upregulated or control HCC cells via tail vein injections for 12 weeks. Arrowheads indicate the tumour. The number of lung metastatic nodules in each mouse is visualized. Mean ± SD, *n* = 5–6, Student's *t*‐test, *P < 0.05. (G) Representative images of the murine lung metastasis model at the indicated times were captured with the In Vivo Fx Pro Imaging System. Signal intensity of the luciferase activity in tumours was quantified as the mean density of photon flux (p·s^−1^·mm^−2^). (H) HE staining of tissue sections showing lung metastatic foci in nude mice xenografted with Tspan5‐upregulated or control tumour cells. The size and number of lung metastatic foci were calculated. Mean ± SD, *n* = 5–6, Student's *t*‐test, *P < 0.05, **P < 0.01. Scale bar: 1000 μm (for MHCC97L) or 400 μm (for BEL7402).

To understand the function of Tspan5 in tumour metastasis of HCC, we first examined the endogenous expression of Tspan5 in a variety of HCC cell lines by qRT‐PCR and western blotting (Fig. S2). We then manipulated Tspan5 expression in three representative cell lines by lentivirus transductions. Upregulation of Tspan5 was confirmed in MHCC97L‐Tspan5, Hep3B‐Tspan5 and BEL7402‐Tspan5 cell lines, whereas downregulation of Tspan5 was verified in MHCC97L‐shTspan5 cells by qRT‐PCR and western blotting (Fig. 2A,B). Wound‐healing and Boyden chamber cell migration assays were then performed to investigate the migration capacity of these engineered tumour cells. Compared with each relative control, MHCC97L‐Tspan5, Hep3B‐Tspan5 and BEL7402‐Tspan5 cells increased their abilities of wound healing (Fig. 2C) and Transwell migration (Fig. 2D), whereas MHCC97L‐shTspan5 cells decreased their wound healing and migration capacities (Fig. 2C,D). Thus, the results indicate that Tspan5 promote HCC cell migration *in vitro*.

We then performed xenograft experiments by injection of tumour cells into the tail vein of nude mouse to verify the *in vitro* findings via lung metastasis models. After 12 weeks, examination of metastatic foci in mouse lungs showed that pulmonary metastasis rates were significantly increased for both MHCC97L‐Tspan5 and BEL7402‐Tspan5 cells compared with that of MHCC97L‐control and BEL7402‐control cells, respectively. Consistently, metastasis rates of MHCC97L‐shTspan5 cells were markedly decreased compared with that of MHCC97L‐shControl (Fig. 2E). There were more metastatic foci in the Tspan5‐upregulated group than in the control group (Fig. 2F). Living images of xenografted mice showed much higher luciferase activity in Tspan5‐upregulated group than that of control group (Fig. 2G). Moreover, compared with each relative control, larger metastatic foci were observed in the Tspan5‐upregulated group than in the Tspan5‐downregulated group after HE staining on lung tissue sections (Fig. 2H). These results indicate that Tspan5 enhances HCC metastasis *in vivo*.

### 3.3 Tspan5 enhances EMT of HCC cells

**Fig. 3:**
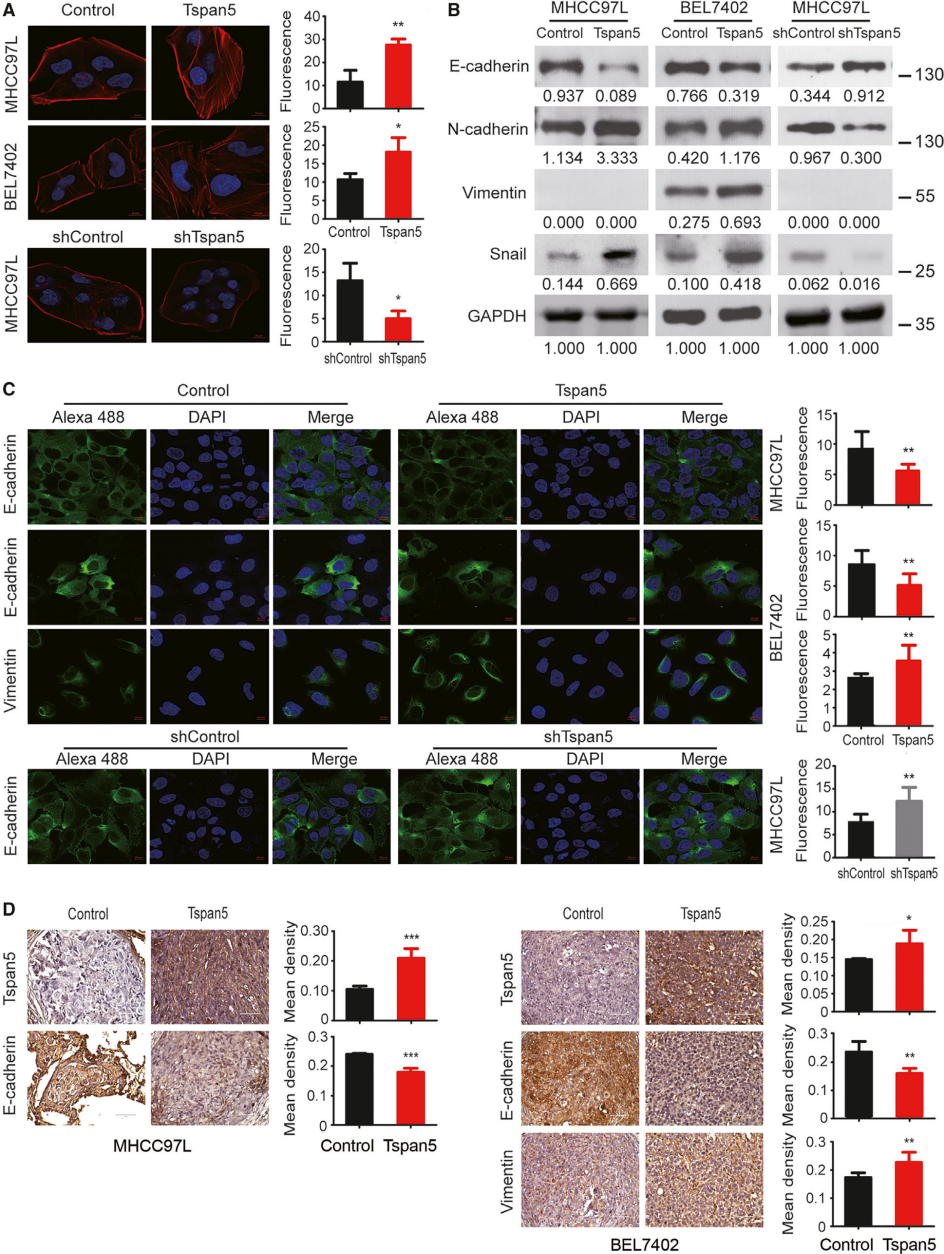
Tspan5 promotes EMT of HCC cells. (A) Tspan5 triggers the rearrangement of actin cytoskeleton. Stress fibres and actin filaments were visualized by Rhodamine‐phalloidin staining (red) in HCC cells transduced with lentivirus containing Tspan5/shTspan5‐encoding vector or empty/scramble RNA vector (Control/shControl). Nuclei were stained with DAPI (blue). Upregulation of Tspan5 significantly increased F‐actin expression (red) and actin stress fibres throughout the Tspan5‐upregulating cells compared with the control cells in which the actin bundles were predominantly localized underneath cell membranes. 400× magnification, scale bar: 10 μm. Mean ± SD, *n* = 3, Student's *t*‐test; *P < 0.05, **P < 0.01. (B) Western blotting showing regulation of the expression of EMT markers, E‐cadherin, N‐cadherin, vimentin and Snail by Tspan5. GAPDH was used as a protein loading control. Numbers indicating relative protein ratio measured by image j software and normalized to GAPDH. Vimentin was undetectable in MHCC97L cells. (C) Representative IF images showing regulation of the expressions of E‐cadherin and vimentin by Tspan5. 630× magnifications, scale bar: 10 μm. Quantification of the protein expression was performed by Zeiss zen microscope software. Mean ± SD, *n* = 9, Student's *t*‐test; **P < 0.01. (D) Representative IHC images of Tspan5, E‐cadherin and vimentin expressions in xenograft sections of metastatic foci in mouse lungs. 600× magnifications, scale bar: 50 μm. Mean ± SD, *n* = 4–6, Student's *t*‐test; *P < 0.05, **P < 0.01, ***P < 0.001.

In all three Tspan5‐upregulated cell lines (e.g. MHCC97L‐Tspan5, BEL7402‐Tspan5 and Hep3B‐Tspan5) we observed clear spindle‐like morphologies, whereas each control cell line (e.g., MHCC97L‐control, BEL7402‐control and Hep3B‐control) displayed cobblestone‐like appearance (Fig. S3A). F‐actin staining of tumour cells with Rhodamine‐phalloidin revealed that upregulation of Tspan5 significantly increased the expression of F‐actin and propelled actin cytoskeleton rearrangement in both MHCC97L‐Tspan5 and BEL7402‐Tspan5 cells as compared with that of the control cells. Typical cortical actin cytoskeleton was clearly observed in the control cells, whereas actin stress fibres throughout the cells, characteristic of migrating mesenchymal cells, were explicitly visualized in both MHCC97L‐Tspan5 and BEL7402‐Tspan5 cells. Consistently, downregulation of Tspan5 had the opposite impact (Fig. 3A). Western blotting demonstrated that upregulation of Tspan5 strikingly decreased the expression of E‐cadherin but increased the expression of N‐cadherin, vimentin and Snail in MHCC97L‐Tspan5, BEL7402‐Tspan5 and Hep3B‐Tspan5 cells as compared with the relative control cells (Fig. 3B, Fig. S3B,C); downregulation of Tspan5, on the other hand, increased expression of E‐cadherin but reduced the expression of N‐cadherin and Snail in MHCC97L‐shTspan5 cells as compared with MHCC97L‐shControl cells. Consistently, immunofluorescence (IF) assays produced similar results in all tested cell lines, that is, that upregulation of Tspan5 decreased E‐cadherin expression but increased vimentin expression in both MHCC97L and BEL7402 cell lines, whereas downregulation of Tspan5 increased the expression of E‐cadherin in MHCC97L cells (Fig. 3C). IHC staining on tumour sections of metastasized lungs demonstrated that upregulation of Tspan5 significantly reduced the expression of E‐cadherin but increased the expression of vimentin in MHCC97L and BEL7402 tumours (Fig. 3D). These results indicate that Tspan5 promotes EMT of HCC cells.

### 3.4 Tspan5 activates Notch signalling in HCC cells

**Fig. 4:**
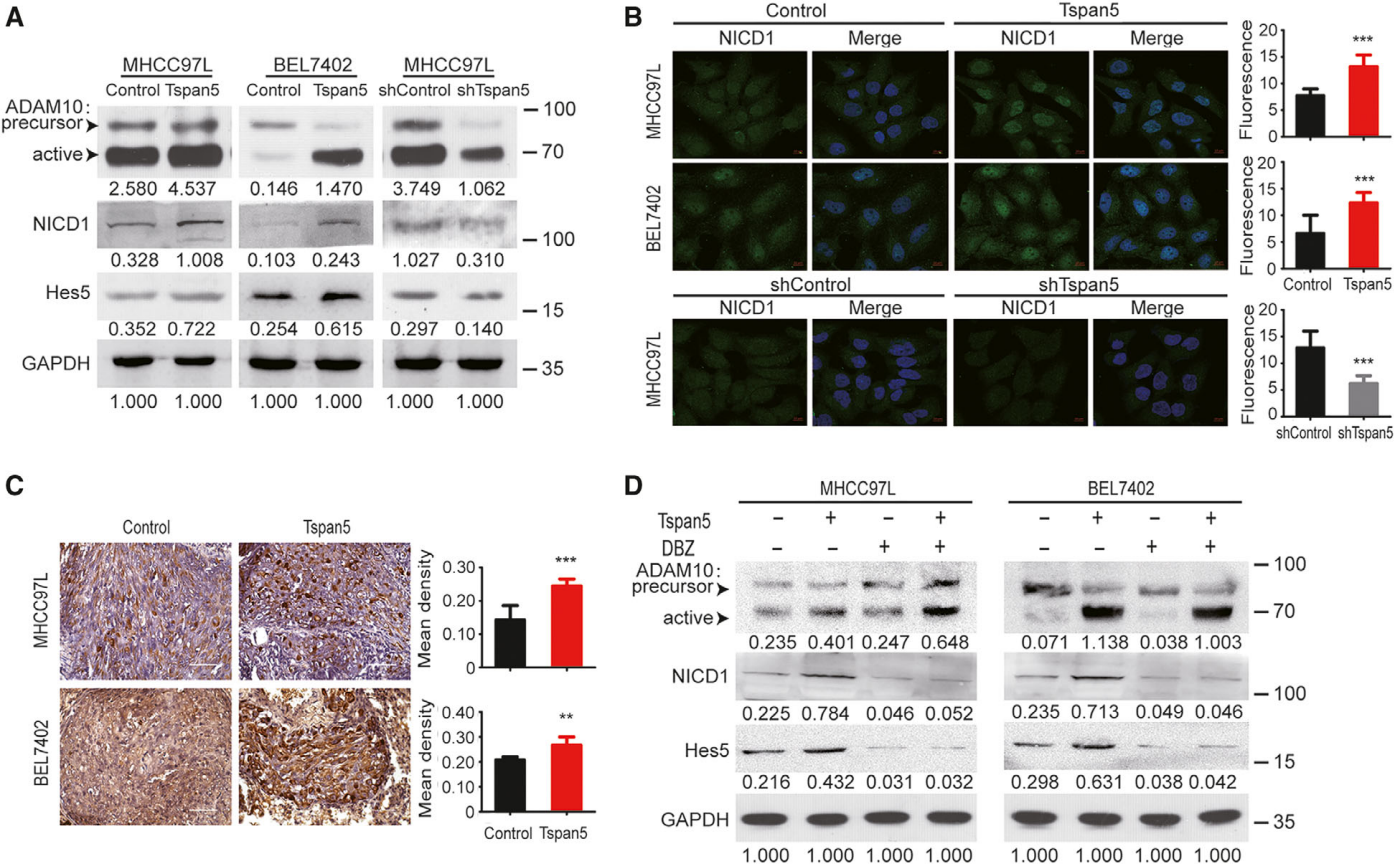
Tspan5 activates Notch signalling in HCC cells. (A) Western blotting showing that Tspan5 regulates the expressions of ADAM10, NICD1 (Val1744) and Hes5 in tumour cells. GAPDH was used as the protein loading control. Numbers indicate relative protein ratio measured by image j software and normalized to GAPDH. (B) Representative images of IF for NICD1 showing that Tspan5 affects the expression and nuclear translocation of NICD1. 630× magnifications, scale bar: 10 μm. Quantification of the protein expression was performed by Zeiss zen microscope software. Mean ± SD, *n* = 9, Student's *t*‐test; ***P < 0.001. (C) Representative IHC images of NICD1 expression and nuclear localization in xenograft sections of metastatic foci in mouse lungs. 600× magnifications, scale bar: 50 μm. Mean ± SD, *n* = 4–6, Student's *t*‐test; **P < 0.01, ***P < 0.001. (D) Western blotting showed that DBZ treatment (60 nm for BEL7402 and 4 nm for MHCC97L) does not significantly affect the expression of ADAM10 but fully abolishes the upregulation of NICD1 (Val1744) and Hes5 by Tspan5. GAPDH was used as a protein loading control. Numbers indicate relative protein ratio measured by image j software and normalized to GAPDH.

We assessed the effect of Tspan5 on Notch signalling in HCC cells. Western blotting demonstrated that upregulation of Tspan5 enhanced the expression of active ADAM10, NICD1 (activated Notch1 intracellular domain) and Hes5 (Hes family BHLH transcription factor 5) in all MHCC97L‐Tspan5, BEL7402‐Tspan5 and Hep3B‐Tspan5 cell lines, whereas downregulation of Tspan5 reduced the expressions of active ADAM10, NICD1 and Hes5 in MHCC97L‐shTspan5 cells as compared with the relative controls (Fig. 4A, Fig. S4A,B). IF assays confirmed that upregulation of Tspan5 increased NICD1 expression and nuclear translocation in both MHCC97L‐Tspan5 and BEL7402‐Tspan5 cells, whereas downregulation of Tspan5 decreased NICD1 expression and nuclear translocation in MHCC97L‐shTspan5 cells (Fig. 4B). Moreover, IHC staining on xenograft sections of metastatic foci in mouse lungs revealed that upregulation of Tspan5 in tumour cells enhanced the expression of NICD1 and nuclear localization *in vivo* as well (Fig. 4C). To verify further the activation of Notch signalling in HCC cells by Tspan5, we treated HCC cells with the γ‐secretase inhibitor, dibenzazepine (DBZ, YO‐01027) for 24 h and then performed western blotting assays. DBZ treatment did not significantly affect ADAM10 expression in either Tspan5‐upregulated and control cells, but fully abolished the enhanced expression of NICD1 and Hes5 by Tspan5 (Fig. 4D, Fig. S4C). These results indicate that Tspan5 enhances the enzymatic maturation of ADAM10 and activates Notch signalling by increased the cleavage of Notch receptor at the S3 site catalyzed by γ‐secretase in HCC cells.

### 3.5 Tspan5 promotes metastasis and EMT through Notch signalling

**Fig. 5:**
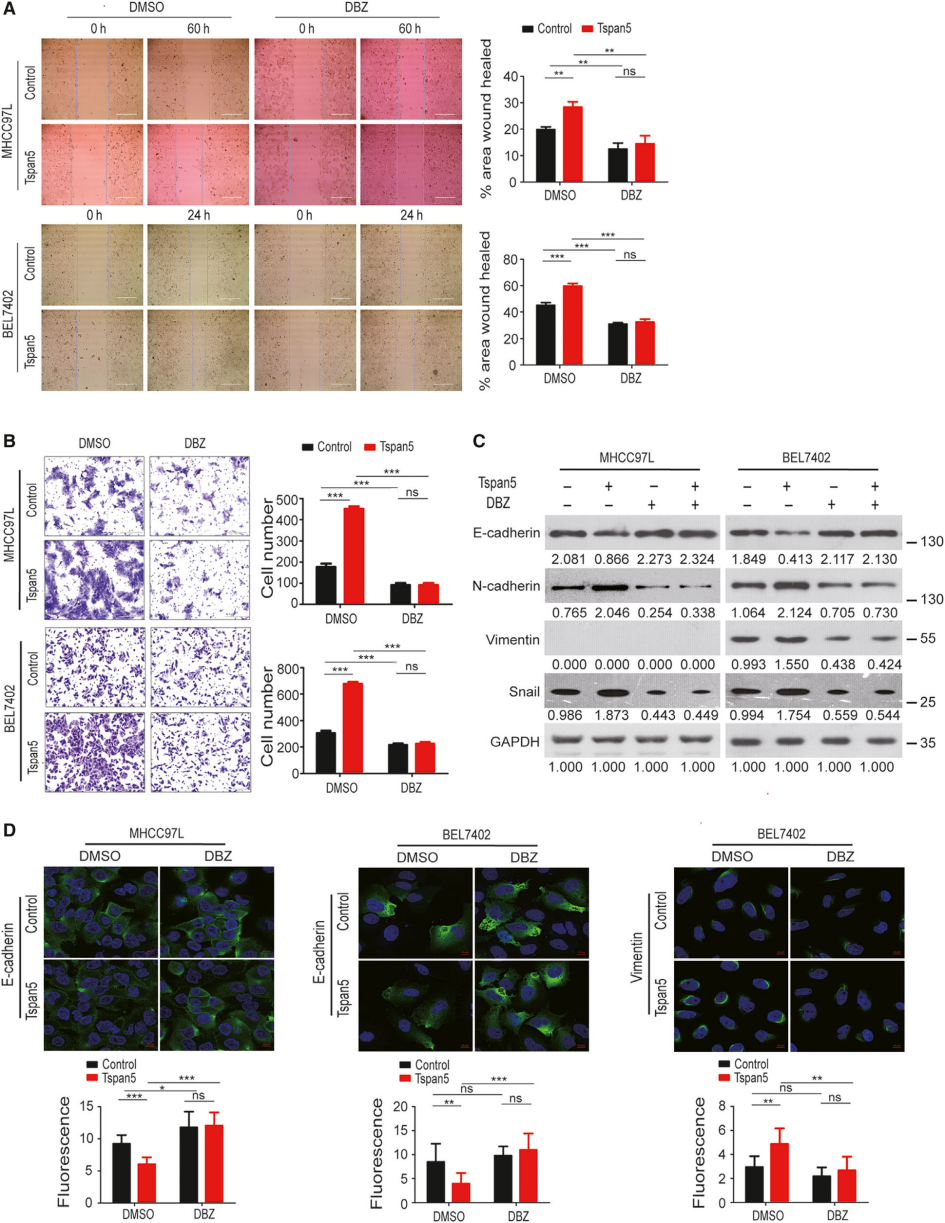
Tspan5 promotes metastasis and EMT by Notch1 signalling in HCC cells. (A) Wound‐healing assays showing that blockade of Notch signalling by DBZ greatly decreases the migration of HCC cells. Mean ± SD, ANOVA test, *n* = 3, **P < 0.01, ***P < 0.001, ns = no significance. Scale bar = 400 μm. (B) Boyden chamber migration assays showing that blockade of Notch signalling by DBZ greatly decreases the migration of HCC cells. Mean ± SD, ANOVA test, *n* = 3, ***P < 0.001, ns = no significance. Scale bar: 200 μm. (C) Western blotting showing that blockade of Notch signalling by DBZ strikingly abolishes the effects of Tspan5 on regulating the expressions of EMT markers, E‐cadherin, N‐cadherin, vimentin and Snail in HCC cells. GAPDH was used as the protein loading control. Numbers indicate relative protein ratio measured by image j software and normalized to GAPDH. (D) IF assays showing that blockade of Notch signalling by DBZ abolishes the effects of Tspan5 on the expression of E‐cadherin and vimentin in HCC cells. 630× magnification, scale bar: 10 μm. Quantification of the protein levels were performed by Zeiss zen microscope software. Mean ± SD, *n* = 9, ANOVA test, *P < 0.05, **P < 0.01, ***P < 0.001, ns = no significance.

We investigated the pathway connection for the function that Tspan5 played in HCC metastasis, EMT and Notch signalling by blockade of Notch signalling in tumour cells with DBZ. Wound‐healing and Boyden chamber cell migration assays revealed that blockade of Notch signalling by DBZ treatment not only abolished the enhanced migration ability conferred by Tspan5 but also further decreased the migration capacity of both MHCC97L and BEL7402 cells (Fig. 5A,B). Western blotting analysis demonstrated that DBZ treatment not only eradicated all effects of Tspan5 on decreasing the expression of E‐cadherin and increasing the expression of N‐cadherin, vimentin and Snail but also further decreased the expression of N‐cadherin, vimentin and Snail as compared with the DMSO control in both MHCC97L and BEL7402 cells (Fig. 5C, Fig. S5). IF assays further confirmed that upregulation of Tspan5 reduced the expression of E‐cadherin and increased the expression of vimentin; DBZ treatment not only eliminated the effects of Tspan5 on reducing the expression of E‐cadherin and increasing the expression of vimentin but also further increased the expression of E‐cadherin in both cell lines (Fig. 5D). These results suggest that Tspan5 promotes HCC metastasis by increasing Notch signalling and EMT process.

### 3.6 Correlation of Tspan5 with key players in Notch signalling and EMT in clinical HCC samples

**Fig. 6:**
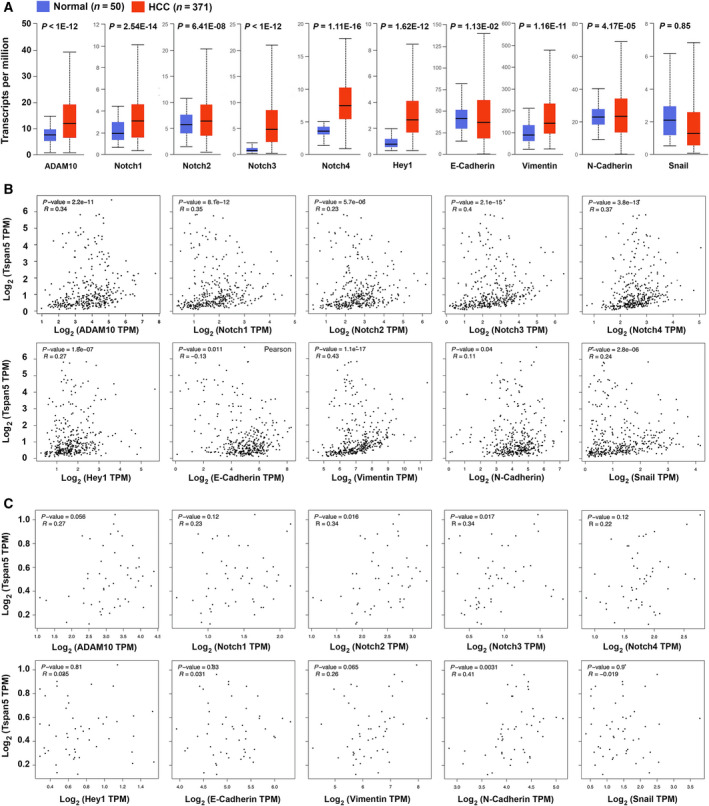
Expression and correlation of key players in Notch signalling and EMT with Tspan5 in clinical HCC samples. (A) The expression of ADAM10, Notch1, Notch2, Notch3, Notch4, Hey1, vimentin and N‐cadherin was significantly upregulated, whereas transcripts of E‐cadherin were significantly downregulated in 371 TCGA tumour tissues versus 50 normal liver tissues of HCC. (B) Correlation analyses of TCGA tumour samples of HCC using the GEPIA server (http://gepia.cancer‐pku.cn/detail.php?clicktag=correlation) demonstrated that Tspan5 expression is highly correlated with all players in Notch signalling and EMT in 371 clinical HCC samples. (C) Correlation analyses of TCGA tumour samples of HCC using the GEPIA server showed that Tspan5 expression is not significantly correlated with ADAM10, Notch1, Notch4, Hey1, E‐cadherin, vimentin or Snail in 50 HCC normal liver tissues.

We further investigated the expression of key players of Notch signalling and EMT in TCGA tumour samples. The expression of ADAM10, Notch1, Notch2, Notch3, Notch4, Hey1, vimentin and N‐cadherin at the transcript level was significantly upregulated, whereas transcripts of E‐cadherin significantly decreased in tumour tissues versus in normal liver tissues of HCC (Fig. 6A). No other significant difference in Snail expression was found between HCC tissues and normal liver tissues. Tspan5 correlation analyses of TCGA tumour samples revealed that Tspan5 expression is positively correlated with the expression of ADAM10, Notch1, Notch2, Notch3, Notch4, Hey1, vimentin, N‐cadherin and Snail, whereas it is negatively correlated with E‐cadherin in HCC tissues (Fig. 6B). Interestingly, such correlations of Tspan5 with ADAM10, Notch1, Notch4, Hey1, vimentin, E‐cadherin and Snail were not found in HCC normal liver tissues (Fig. 6C). Thus, the results substantiate the role of Tspan5 in the regulation of Notch signalling, EMT and tumour metastasis of HCC.

## 4 Discussion

Tspan5 is a member of the tetraspanin subfamily of TspanC8 that comprises Tspan5, Tspan10, Tspan14, Tspan15, Tspan17 and Tspan33. TspanC8 tetraspanins are characterized by the eight cysteine residues in the LEL (other tetraspanins have four, six or seven cysteines instead) [23, 24] and are closely associated with ADAM10 in promoting its intracellular trafficking from the endoplasmic reticulum through the Golgi to different subcellular localizations [23, 24, 25, 34, 35]. Due to the difference of expression repertoires in different cell types and different subcellular localizations in the same cell type, different TspanC8 members can differentially regulate the ADAM10‐mediated cleavage of distinct substrates [34, 36, 37]. Tspan5, Tspan10 and Tspan14 increase Notch signalling, whereas Tspan15 and Tspan33 have the opposite effect [23, 25, 26, 34]. However, the expression repertories or biological functions of TspanC8 members in liver, particularly in HCC, are unknown.

In this study, we focused on the biological function and clinical significance of Tspan5 in HCC. We demonstrated for the first time that Tspan5 is significantly upregulated in HCC and associated with tumour invasive depth, clinical stage and poor overall survival of patients, particularly those with tumour vascular invasions, heavy alcohol consumption or hepatitis virus infections. It is well known that HCC incidence is highly associated with heavy alcohol consumption and hepatitis virus infections [3]. Thus, we hypothesized Tspan5 may be involved in tumour metastasis and disease progression of HCC patients. To investigate the role of Tspan5 in tumour metastasis of HCC, we modulated the expression of Tspan5 in HCC cells by lentivirus‐mediated transductions. We found that upregulation of Tspan5 significantly promotes the wound healing and migration of HCC cells *in vitro* and tumour metastasis of HCC *in vivo*. In agreement with the upregulation result, Tspan5 downregulation dramatically inhibited the wound healing and migration of HCC cells *in vitro* and the tumour metastasis *in vivo*. We therefore conclude that Tspan5 may regulate disease progression by increasing cell migration and tumour metastasis of HCC.

Epithelial–mesenchymal transition, a fundamental biological process that enables polarized epithelial cells to convert mesenchymal‐like cells, rearranges actin cytoskeleton and confers cell migration and invasion [38], plays crucial roles in the stem cell properties, cell senescence and apoptosis as well as tissue and organ repair [39]. It is reported that EMT plays a key role in HCC development, and many molecules and pathways are involved in this [40]. However, no previous study has shown the connection of Tspan5 or other TspanC8 members with EMT. We have found for the first time that Tspan5 upregulation facilitates the morphological conversion of tumour cells from the epithelial‐like to more mesenchymal‐like phenotypes, increases the expression of F‐actin and actin cytoskeleton rearrangement from the actin bundles underneath cell membranes to dynamic structures of actin fibres throughout the cells, reduces the expression of E‐cadherin (an important caretaker for the epithelial phenotype) and increases the expression of N‐cadherin and vimentin (two goalkeepers for the mesenchymal phenotype). Consistently, Tspan5 downregulation produces opposite impacts *in vitro*. These findings on the regulation of E‐cadherin and vimentin expressions by Tspan5 were verified by IHC on tumour sections metastasized in mouse lungs. We conclude that Tspan5 increases the cell migration and tumour metastasis by impelling EMT of HCC cells.

ADAM10 is a member of the superfamily of Zn^2+^‐dependent transmembrane disintegrin and metalloproteases that are responsible for a significant proportion of transmembrane protein shedding [41]. ADAM10 is ubiquitously expressed in mammalian cells and has more than 40 protein substrates, of which Notch receptor is one of the most important substrates [42]. ADAM10 cleaves Notch ectodomain at the S2 site [43, 44], resulting in a conformational change of Notch, and enables Notch to be recognized and cleaved by the γ‐secretase complex at the S3 site. The γ‐secretase cleavage releases Notch intracellular domain (NICD) from the cell membrane. NICD is then translocated to the nucleus and activates the transcription of Notch target genes, including those encoding Hes family of transcript factors [43, 45]. It is known that Notch signalling plays crucial roles in liver disease [46]. However, which member of Notch receptors plays the predominant role in HCC is controversial [47, 48, 49]. Notch1 has been more extensively studied [49], showing an important role in tumour metastasis when expressed in tumour‐associated endothelial cells [50]. To explore the specific role of Tspan5 in regulation of Notch signalling in HCC, we determined the expression of key players in Notch signalling after modulating the expression of Tspan5 in tumour cells. We found that Tspan5 upregulation enhanced the expression of active ADAM10, NICD1 and Hes5 as well as the nuclear localization of NICD1, whereas Tspan5 downregulation decreased the expressions of active ADAM10, NICD1 and Hes5 in HCC cells and in xenograft sections of metastatic foci in mouse lungs. Treatment of HCC cells with the γ‐secretase inhibitor, DBZ, did not affect the expression of active ADAM10 but fully eradicated the expressions of NICD1 and Hes5 enhanced by Tspan5 in HCC cells. Therefore, our findings indicate that Tspan5 enhances the enzymatic maturation of ADAM10 and promotes Notch signalling via increasing the cleavage of Notch S3 site catalyzed by the γ‐secretase complex in HCC.

We speculated that Tspan5 promotes the metastasis and EMT through activating Notch signalling. To test this hypothesis, we assessed the wound healing and Transwell migration capacity of Tspan5‐engineered tumour cells as well as the expression of EMT markers upon blockade of Notch signalling by the γ‐secretase inhibitor. DBZ treatment not only abolished the wound healing and migration ability of tumour cells enhanced by Tspan5 but also further decreased the wound healing and migration capacity of all HCC cells. Similarly, DBZ not only fully eliminated all effects of Tspan5 on decreasing the expression of E‐cadherin and increasing the expressions of N‐cadherin, vimentin and Snail, but also further enhanced the epithelial marker expression and reduced the mesenchymal marker expressions in all cells. The more profound effects on the migration and EMT of tumour cells by DBZ than by Tspan5 itself were not surprising, as the γ‐secretase complex would act on all four Notch receptors (Notch1–4) and perhaps other proteins in HCC cells [47, 48, 49]. Thus, our findings indicate that Tspan5 facilitates the tumour metastasis and EMT by activation of Notch signalling in HCC.

To verify the results from *in vitro* and *in vivo* experiments, we further investigated the expression and correlation of key players in Notch signalling and EMT with Tspan5 in in TCGA clinical samples of HCC. We revealed that the expression of ADAM10, Notch1, Notch2, Notch3, Notch4, Hey1, vimentin and N‐cadherin is significantly upregulated, whereas the expression of E‐cadherin is downregulated in HCC tissues, consistent with the expression of Tspan5. Importantly, Tspan5 is significantly correlated with all these elements, greatly substantiating the role of Tspan5 in the regulation of Notch signalling, EMT and tumour metastasis of HCC. More importantly, most such correlations (7/10) do not exist in HCC normal tissues, suggesting they are HCC tumour‐specific. Therefore, we speculate that specific blockades of Tspan5‐induced Notch signalling and EMT may produce significant clinical benefits for HCC patients who have had elevated activities of Tspan5, Notch signalling and EMT in their primary tumours.

## 5 Conclusions

**Fig. 7:**
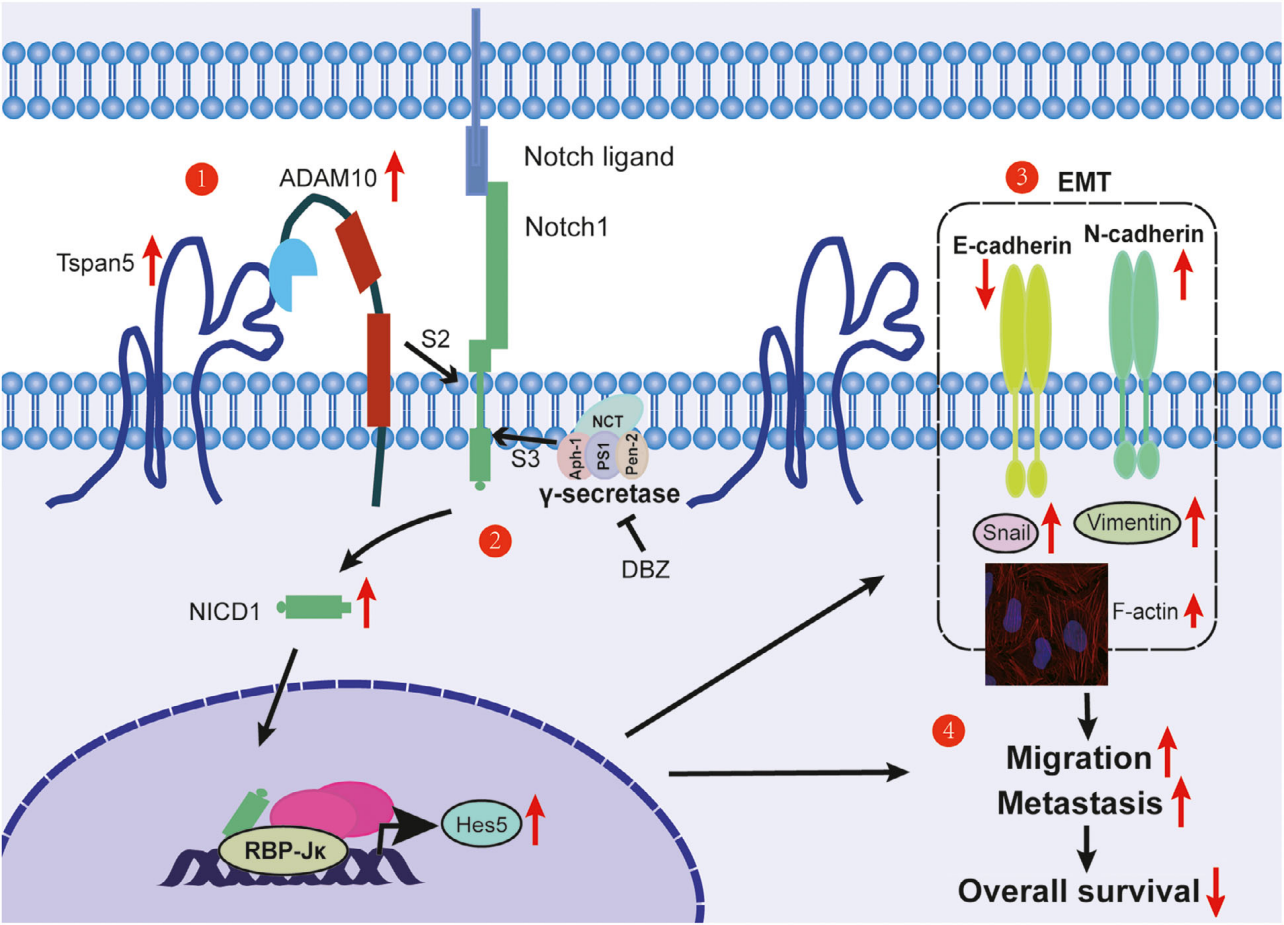
Schematic diagram shows the function of Tspan5 in the regulation of Notch signalling, EMT and tumour metastasis in HCC. Key results are summarized in the working model. (1) Tspan5 interacts with ADAM10 and increases the activity of ADAM10 that cleaves Notch1 receptor at the S2 site. (2) Tspan5 activates Notch signalling by increasing the cleavage of Notch1 receptor at the S3 site catalysed by the γ‐secretase complex for the release of NICD from cell membrane. NICD is then translocated to the nucleus, where it activates the transcription of Notch‐targeted genes including Hes5. (3) Tspan5 enhances EMT and actin skeleton rearrangement of HCC cells by regulation of the expression of the epithelial marker, E‐cadherin and mesenchymal markers, N‐cadherin, vimentin and Snail. (4) Tspan5 promotes the migration and tumour metastasis, thereby impelling the disease progression and overall survival of HCC patients.

We have demonstrated for the first time that Tspan5 is significantly upregulated and closely associated with tumour invasion, clinical stage and overall survival of HCC patients. Modulation of Tspan5 in HCC cells has shown that Tspan5 promotes the cell migration and tumour metastasis of HCC by increasing the enzymatic maturation of ADAM10, activating Notch signalling via increasing the cleavage of Notch S3 site catalyzed by γ‐secretase, and enhancing Notch‐dependent EMT and actin skeleton rearrangement of HCC cells. In HCC clinical specimens, Tspan5 is highly correlated with the expression of all key players in Notch signalling and EMT process, highlighting the role of Tspan5 in tumour metastasis of HCC by regulation of Notch signalling and EMT (Fig. 7). Our results provide new insights into the mechanism of tumour metastasis of HCC and rationales for the development of novel intervention strategies against HCC.

## Conflicts of interest

The authors declare no conflicts of interest.

## Author contributions

QX, SW and J‐LL conceptualized the study and designed the research. QX, SW, HG, PH, HD, YG, ND and WN performed research. QX, SW and J‐LL analysed data. TL provided administrative support. ML, YW and JL‐L supervised research. QX drafted the manuscript. J‐LL wrote the paper. All authors approved the manuscript.

## Data availability

The data that support the findings of this study are available from the corresponding author (jlilab1971@yahoo.co.uk) upon reasonable request.

## Supporting information


**Fig. S1.** Expression and association of Tspan5 in liver tumour tissues with pathological grade and clinical stage of HCC patients. (A) IHC staining for Tspan5 protein expressed in HCC tissue arrays with 139 clinical HCC samples showed that Tspan5 expression is mainly located on cell membranes and in cytoplasm. Representative images for high and low expression of Tspan5 at 100× (400 μm) and 600× (50 μm) magnification. (B) Expression of Tspan5 in HCC tissue was 1.2~2.6‐fold higher than that of normal liver tissues in Oncomine datasets (*P* < 0.001). FC, fold change for the expression of Tspan5 in HCC tissues versus normal liver tissues. Student's *t*‐test, *n* = number of patients. The error bars indicate maximum and minimum values, respectively. (C) Expression of Tspan5 was gradually increased with pathological tumour grades of HCC in UALCAN database (http://ualcan.path.uab.edu/cgi‐bin/TCGAExResultNew2.pl?genenam=TSPAN5&ctype=LIHC). ANOVA test, *n* = number of patients, *P < 0.05, **P < 0.01, ****P < 0.0001. The error bars indicate maximum and minimum values, respectively.

**Fig. S2.** Basal expression levels of Tspan5 in hepatoma cell lines. (A) The expression of Tspan5 transcripts in human normal hepatocyte (HL7702) and numerous hepatoma cell lines was determined by qRT‐PCR. (B) The expression of Tspan5 protein in human normal hepatocyte (HL7702) and numerous hepatoma cell lines was evaluated by western blotting. GAPDH acted as a protein loading control. Numbers indicate relative protein ratio measured by imagej software and normalized to GAPDH.

**Fig. S3.** Tspan5 promotes EMT of Hep3B cells. (A) Representative phase‐contrast images showing spindle‐like morphologies of MHCC97L‐Tspan5, BEL7402‐Tspan5 and Hep3B‐Tspan5 cell lines, and cobblestone‐like appearance of each relative control cell line. 400× magnifications, scale bar: 100 μm. (B) Western blotting showing upregulation of Tspan5 decreases the expression of E‐cadherin but increases the expression of N‐cadherin, vimentin and Snail in Hep3B cells. GAPDH acted as a protein loading control. Numbers indicate relative protein ratio measured by imagej software and normalized to GAPDH. (C) Quantification of western blotting bands in Figure 3B. Mean ± SD, *n* = 3, Student's *t*‐test, *P < 0.05, **P < 0.01, ***P < 0.001.

**Fig. S4.** Upregulation of Tspan5 activates Notch signalling in Hep3B cells. (A) Quantification of western blotting bands in Figure 4A. Mean ± SD, *n* = 3, Student's *t*‐test, *P < 0.05, **P < 0.01, ns = no significance. (B) Upregulation of Tspan5 increases the expression of active ADAM10, activated Notch1 (Val1744) (NICD1) and Hes5 in Hep3B cells. GAPDH acted as a protein loading control. Numbers indicate relative protein ratio measured by imagej software and normalized to GAPDH. (C) Quantification of western blotting bands in Figure 4D. Mean ± SD, *n* = 3, ANOVA test, *P < 0.05, **P < 0.01, ***P < 0.001, ns = no significance.

**Fig. S5.** Quantification of western blotting bands in Figure 5C. Mean ± SD, *n* = 3, ANOVA test, *P < 0.05, **P < 0.01, ***P < 0.001, ns = no significance.
