# Supplementary figures and images for: Tspan5 promotes epithelial–mesenchymal transition and tumour metastasis of hepatocellular carcinoma by activating Notch signalling
Source: Mol Oncol. 2021 Jul 27;15(11):3184–202. doi: 10.1002/1878-0261.12980 (PMC8564648; doi:10.1002/1878-0261.12980)

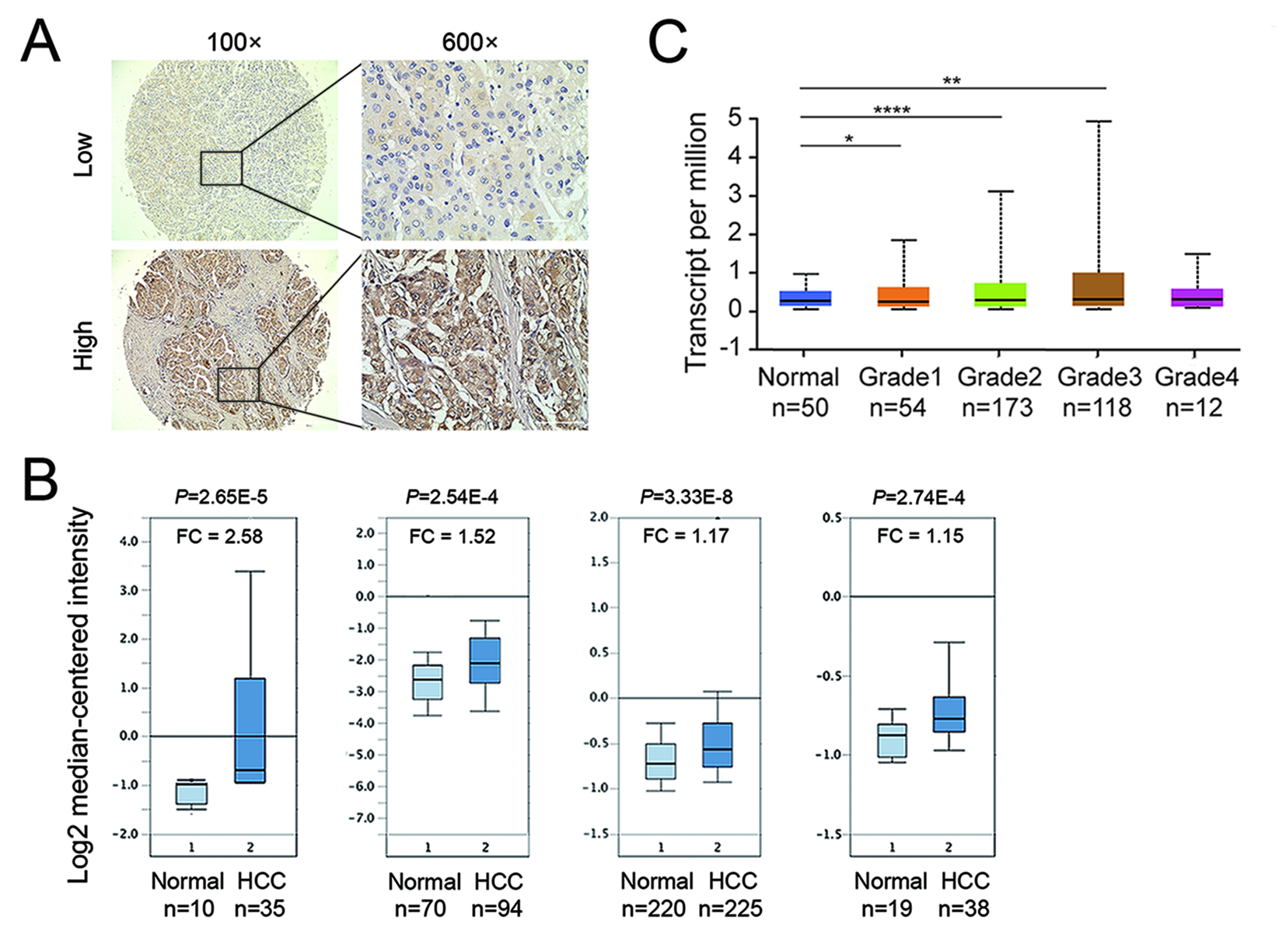

Supplement: Supplementary file 1 — Fig. S1. Expression and association of Tspan5 in liver tumour tissues with pathological grade and clinical stage of HCC patients. (A) IHC staining for Tspan5 protein expressed in HCC tissue arrays with 139 clinical HCC samples showed that Tspan5 expression is mainly located on cell membranes and in cytoplasm. Representative images for high and low expression of Tspan5 at 100× (400 μm) and 600× (50 μm) magnification. (B) Expression of Tspan5 in HCC tissue was 1.2~2.6‐fold higher than that of normal liver tissues in Oncomine datasets (P < 0.001). FC, fold change for the expression of Tspan5 in HCC tissues versus normal liver tissues. Student's t‐test, n = number of patients. The error bars indicate maximum and minimum values, respectively. (C) Expression of Tspan5 was gradually increased with pathological tumour grades of HCC in UALCAN database (http://ualcan.path.uab.edu/cgi‐bin/TCGAExResultNew2.pl?genenam=TSPAN5&ctype=LIHC). ANOVA test, n = number of patients, *P < 0.05, **P < 0.01, ****P < 0.0001. The error bars indicate maximum and minimum values, respectively. [file MOL2-15-3184-s002.tif]

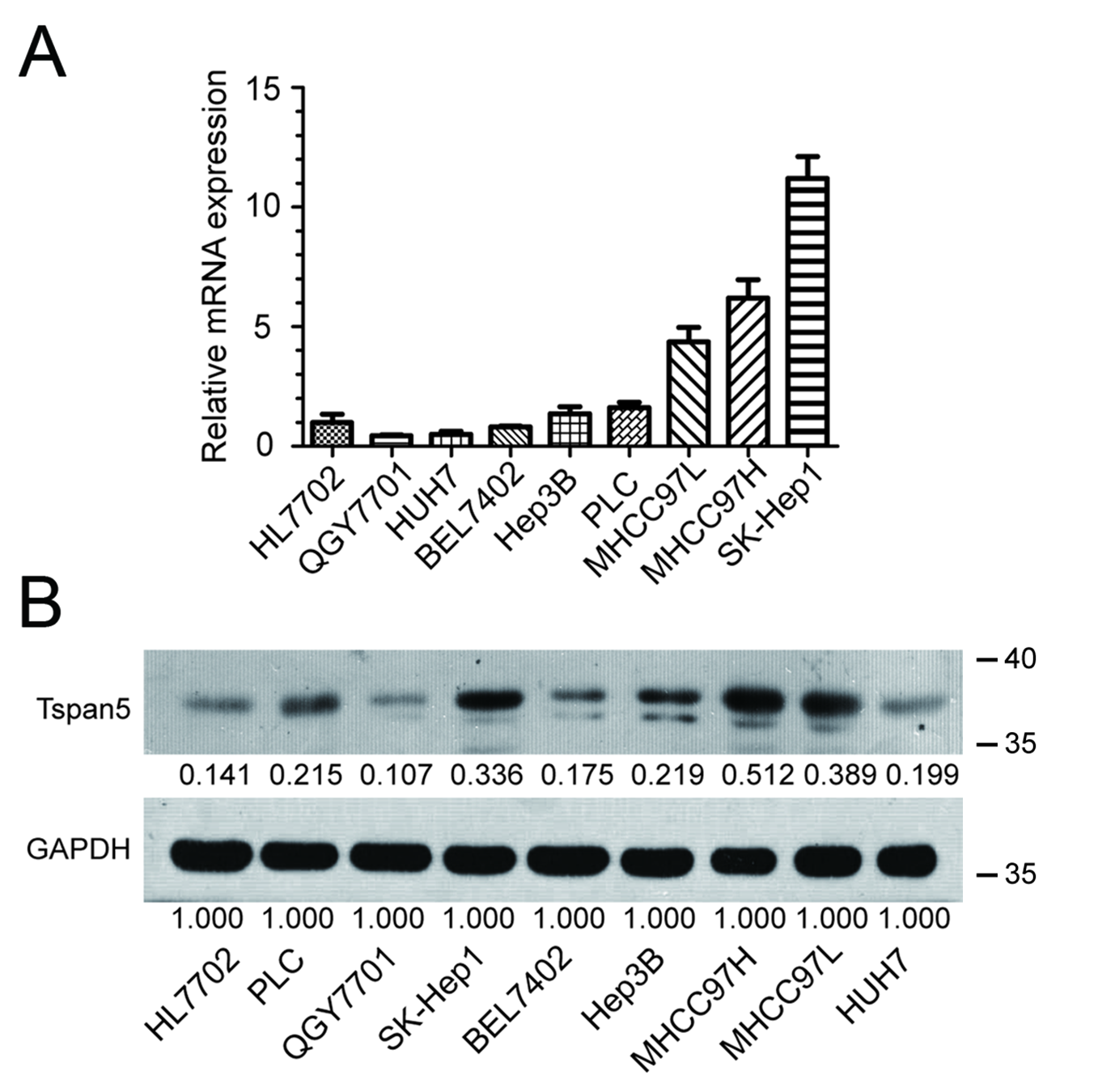

Supplement: Supplementary file 2 — Fig. S2. Basal expression levels of Tspan5 in hepatoma cell lines. (A) The expression of Tspan5 transcripts in human normal hepatocyte (HL7702) and numerous hepatoma cell lines was determined by qRT‐PCR. (B) The expression of Tspan5 protein in human normal hepatocyte (HL7702) and numerous hepatoma cell lines was evaluated by western blotting. GAPDH acted as a protein loading control. Numbers indicate relative protein ratio measured by imagej software and normalized to GAPDH. [file MOL2-15-3184-s001.tif]

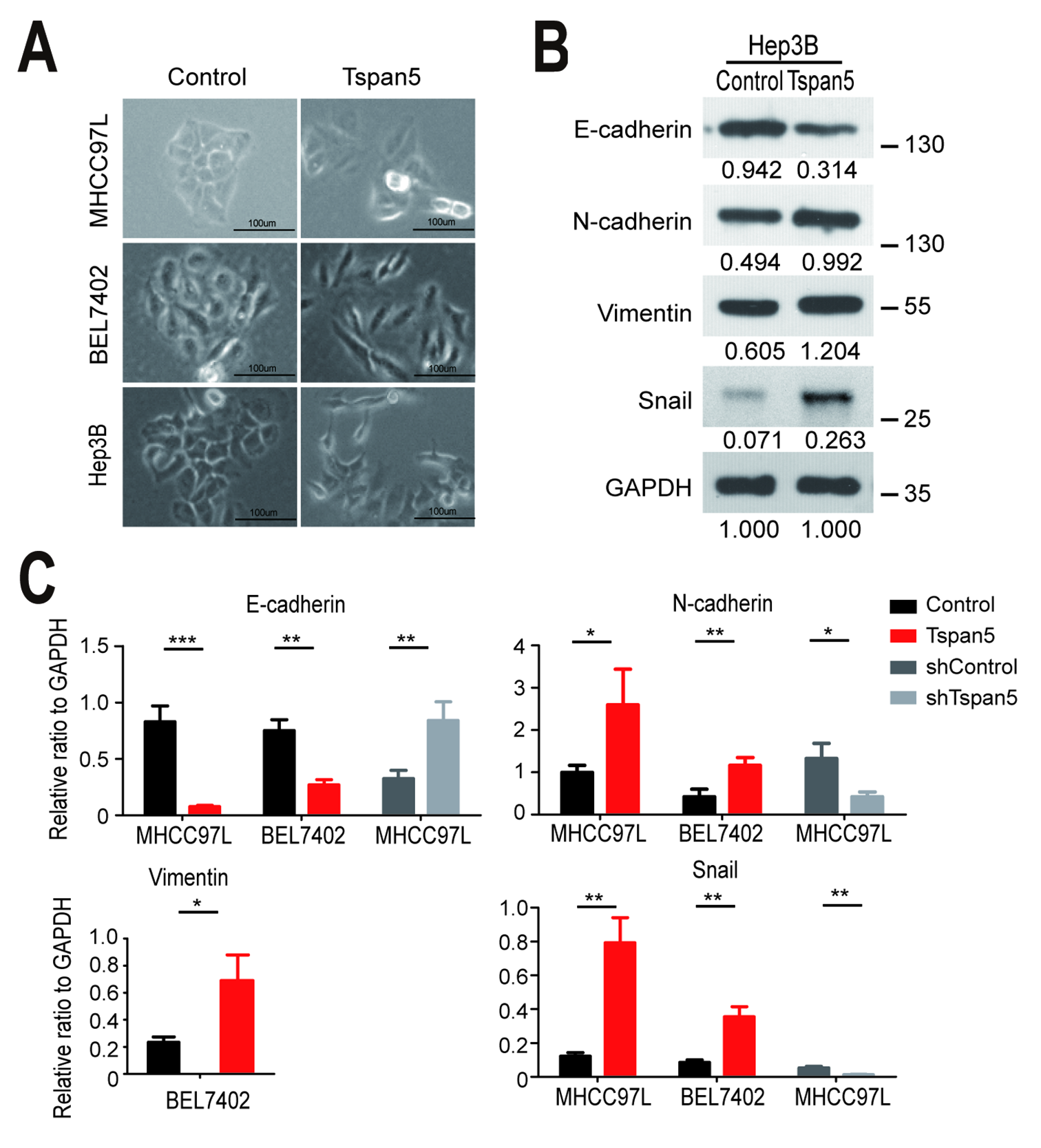

Supplement: Supplementary file 3 — Fig. S3. Tspan5 promotes EMT of Hep3B cells. (A) Representative phase‐contrast images showing spindle‐like morphologies of MHCC97L‐Tspan5, BEL7402‐Tspan5 and Hep3B‐Tspan5 cell lines, and cobblestone‐like appearance of each relative control cell line. 400× magnifications, scale bar: 100 μm. (B) Western blotting showing upregulation of Tspan5 decreases the expression of E‐cadherin but increases the expression of N‐cadherin, vimentin and Snail in Hep3B cells. GAPDH acted as a protein loading control. Numbers indicate relative protein ratio measured by imagej software and normalized to GAPDH. (C) Quantification of western blotting bands in Figure 3B. Mean ± SD, n = 3, Student's t‐test, *P < 0.05, **P < 0.01, ***P < 0.001. [file MOL2-15-3184-s005.tif]

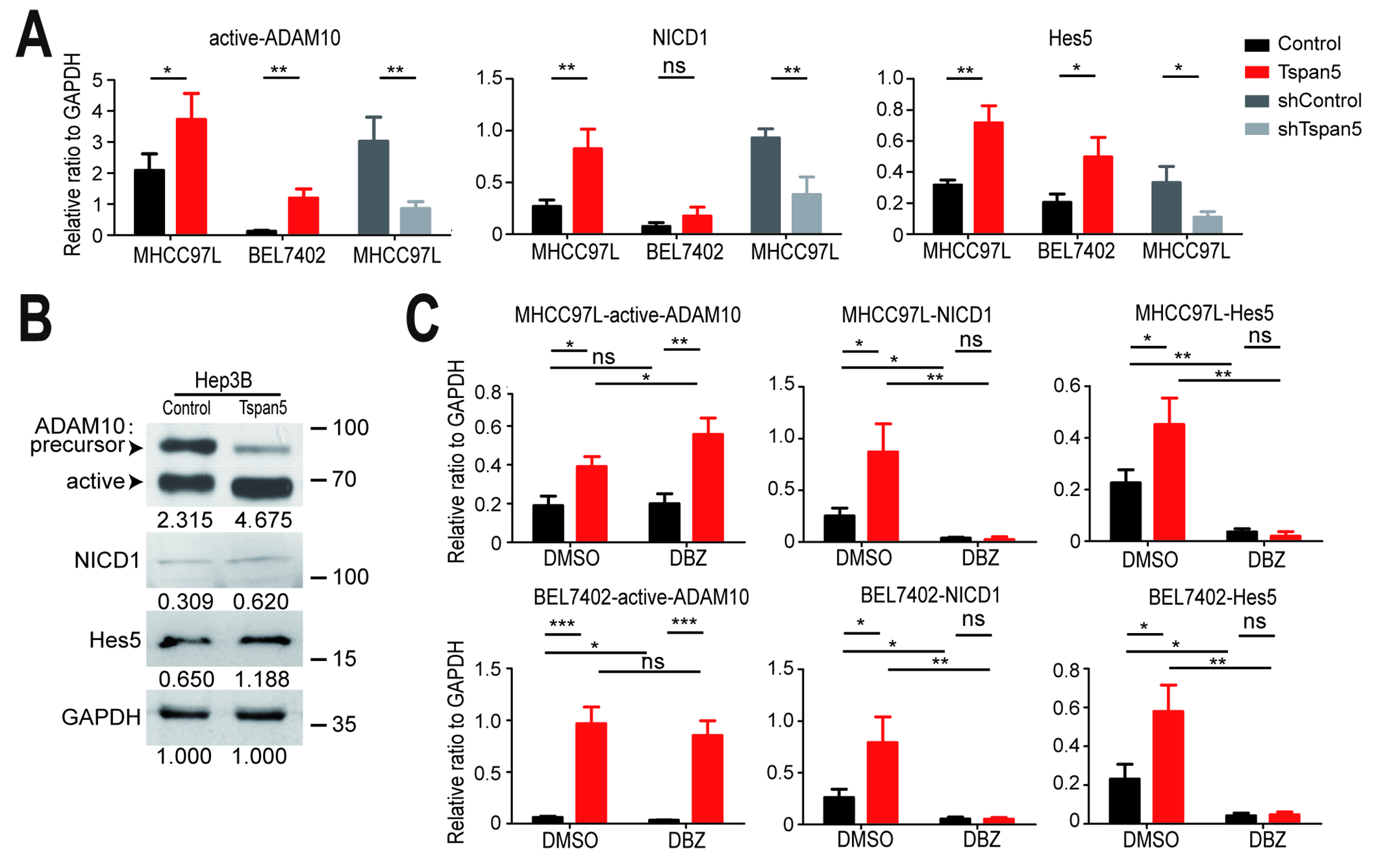

Supplement: Supplementary file 4 — Fig. S4. Upregulation of Tspan5 activates Notch signalling in Hep3B cells. (A) Quantification of western blotting bands in Figure 4A. Mean ± SD, n = 3, Student's t‐test, *P < 0.05, **P < 0.01, ns = no significance. (B) Upregulation of Tspan5 increases the expression of active ADAM10, activated Notch1 (Val1744) (NICD1) and Hes5 in Hep3B cells. GAPDH acted as a protein loading control. Numbers indicate relative protein ratio measured by imagej software and normalized to GAPDH. (C) Quantification of western blotting bands in Figure 4D. Mean ± SD, n = 3, ANOVA test, *P < 0.05, **P < 0.01, ***P < 0.001, ns = no significance. [file MOL2-15-3184-s004.tif]

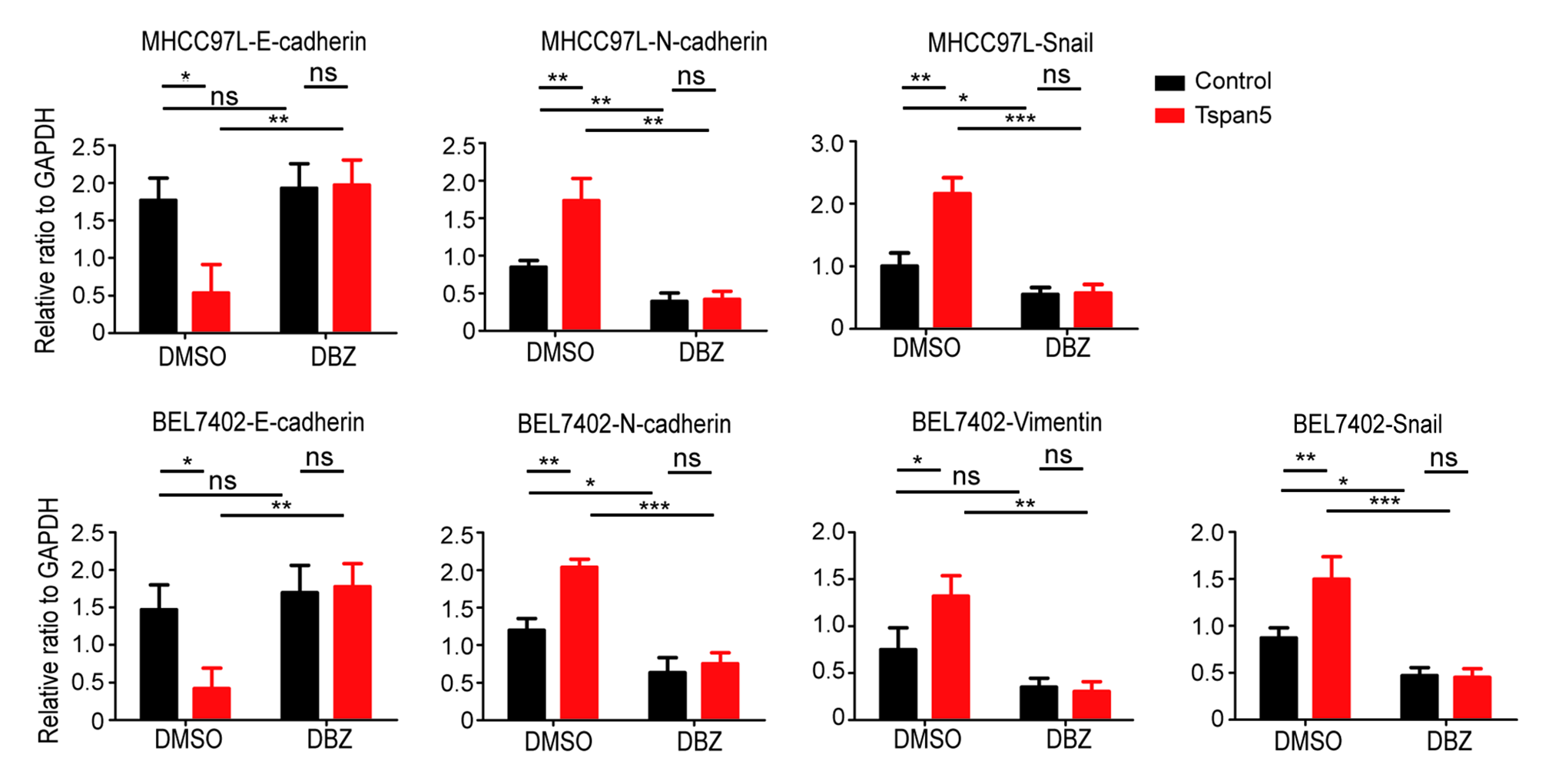

Supplement: Supplementary file 5 — Fig. S5. Quantification of western blotting bands in Figure 5C. Mean ± SD, n = 3, ANOVA test, *P < 0.05, **P < 0.01, ***P < 0.001, ns = no significance. [file MOL2-15-3184-s003.tif]
